# LKB1 loss cooperating with BRAF V600E promotes melanoma cell invasion and migration by up-regulation MMP-2 via PI3K/Akt/mTOR pathway

**DOI:** 10.18632/oncotarget.22943

**Published:** 2017-12-05

**Authors:** Weiming Zhang, Li Yin, Guoxin Song, Xue Han, Zhiqiang Yin, Dan Luo

**Affiliations:** ^1^ Department of Pathology, Nanjing Medical University, Nanjing, Jiangsu 210029, P.R. China; ^2^ Department of Dermatology, The First Affiliated Hospital of Nanjing Medical University, Nanjing, Jiangsu 210029, P.R. China

**Keywords:** melanoma, LKB1, BRAF, MMP-2, PI3K/mTOR

## Abstract

The serine/threonine kinase LKB1, act as a tumor suppressor, has been reported in several sporadic cancers. However, how the loss of LKB1 promotes melanoma invasion and metastasis remains incompletely understood. In this study, we inactivated LKB1expression by RNA interference in BRAF mutation and wild type melanoma cells respectively. We found LKB1 inactivation cooperate with BRAF V600E lead to melanoma cells more aggressive by a series of experiments including wound scratch test, Transwell assay. While single alteration, either LKB1 loss or BRAF V600E, fails to enhance melanoma cells invasion ability. Mechanistically, LKB1 loss synergism with BRAF V600E resulted in the activation of the PI3K/Akt/mTOR signaling pathway and significant up-regulation expression of MMP-2. In addition, LKB1 expression in human melanoma tissues was negatively associated with MMP-2 expression in the presence of BRAF V600E. Thus, our findings indicate a probable explanation on LKB1 function as a tumor suppressor in melanoma and a new therapeutic strategy for melanoma by targeting on BRAF and LKB1 together.

## INTRODUCTION

Malignant melanoma (MM) is a type of cancer originated from uncontrolled proliferation of melanocytes. It notes for its high potential to invasion and metastasis, as well as its resistance to conventional radiotherapy and chemotherapy [1, 2]. Recently, intensive efforts are focused on the interaction of genes alterations in melanoma development [3]. v-Raf murine sarcoma viral oncogene homolog B1 (BRAF) is a member of the RAF protein kinase family and an intermediate in the RAS-RAF-MEK-ERK signaling pathway. It has been found mutated in more than 50% of melanomas with the most common type of valine-to glutamic acid substitution at residue 600(V600E) [4]. Although melanoma patients with oncogenic BRAF V600E mutation have poor prognosis, yet its involvement in invasion that is clinically observed in melanoma patients remains unclear. Current knowledge does not think BRAF gene is a direct driver gene in melanoma tumorigenesis, since there exists no difference between BRAF mutation frequency of primary and metastatic melanoma, even benign and dysplastic naevi display the high mutation frequency, it may be only an early event during neoplastic transformation of melanocytes [5]. Additionally, despite of the successes of BRAF inhibitors in therapy of melanoma patients with BRAF mutation, there are only half of these patients demonstrated effective in the process of treatment and almost all patients will be eventually resistant to inhibitors. Overall, these facts indicate that oncogenic mutation of BRAF is insufficient for melanoma transformation and it may probably accompanied by one or more other genes alterations during tumor progression [6, 7]. Actually, several researchers have reported the similar cooperation events in BRAF mutated tumors. For example, expression of BRAF V600E combined with PTEN loss induced the progression of melanoma by activating PI3K-AKT-mTOR signaling pathway [8], and combined treatment with rapamycin and PD325901 led to shrinkage of observed melanomas, while Geoffery et al put forward that BRAF mutation cooperates with NF1 loss to drive melanoma development through the abrogation of oncogene-induced senescence (OIS) [9].

Liver kinase 1 (LKB1), also known as serine threonine kinase 11 (STK11), regulates cell polarity, proliferation, apoptosis, cell cycle progression, DNA damage response and energy metabolism through directly phosphorylation and activation of adenosine monophosphate (AMP)-dependent kinase (AMPK) and other substrates [10, 11]. It is also mutated in Peutz-Jeghers syndrome, which is predisposed to malignant tumors in multiple tissues [12]. In previous study, LKB1 loss has been corroborated as a risk factor for the development of tumor or a sign of poor prognosis [13]. However, the underlying mechanisms for LKB1-mediated tumor progression are still not fully defined. In melanoma, LKB1 loss display that it may promote melanoma invasion by disrupting directional migration toward extracellular matrix or by inducing the SRC family kinase (SFK)-dependent expansion of a CD24^+^ tumor subpopulation [14–16]. In addition, LKB1 loss also was observed concurrently with KRAS oncogenic mutation promoting pancreatic tumorigenesis by suppression of P21-dependent growth arrest and with BRAF V600E inducing lung adenomas progression to lung carcinomas by overcoming senescence [17, 18]. Here, we investigated the role of LKB1 loss in BRAF V600E mutation and BRAF wild type melanoma cells respectively. Unexpectedly, we found that LKB1 inactivation synergism with BRAF V600E significantly promotes melanoma cells invasion and migration by expression of MMP-2.

## RESULTS

### LKB1 loss alone is insufficient to influence the migration and invasive behavior of BRAF wild type MeWo melanoma cell lines

**Figure 1:**
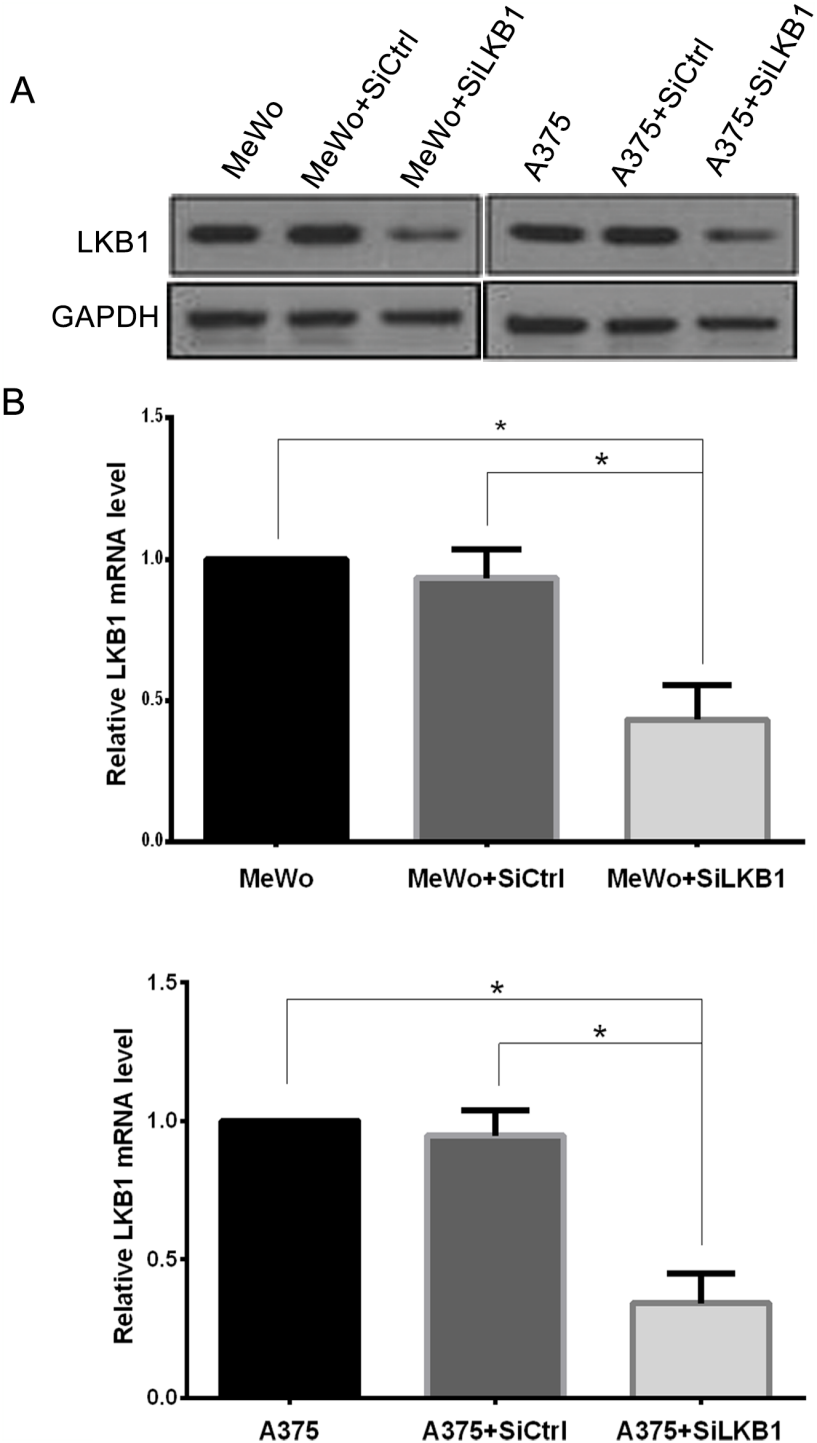
Knockdown of LKB1 in A375 and MeWo cells was confirmed by western blot analysis and qRT-PCR **(A)** LKB1 protein level was detected by western blot analysis. **(B)** LKB1 mRNA level was measured by qRT-PCR. Data are obtained from 3 independent experiments and shown as means±SD of triplicate experiments. ^*^p<0.05.

To investigate the role of LKB1 loss in migration and invasion of melanoma cell lines, LKB1 was knocked down in MeWo human melanoma cells (BRAF wild type) by small interfering RNA targeting on LKB1 (siLKB1), and non-specific siRNA (siCtrl), which has no target in human transcriptome, was used as a negative control. 48 hours after transfection, the efficiency and specificity of LKB1 knockdown were verified by west-blotting and qRT-PCR. As shown in Figure 1, SiLKB1 effectively reduced the expression of LKB1 in MeWo cells, while siCtrl did not affect the expression of LKB1.

**Figure 2:**
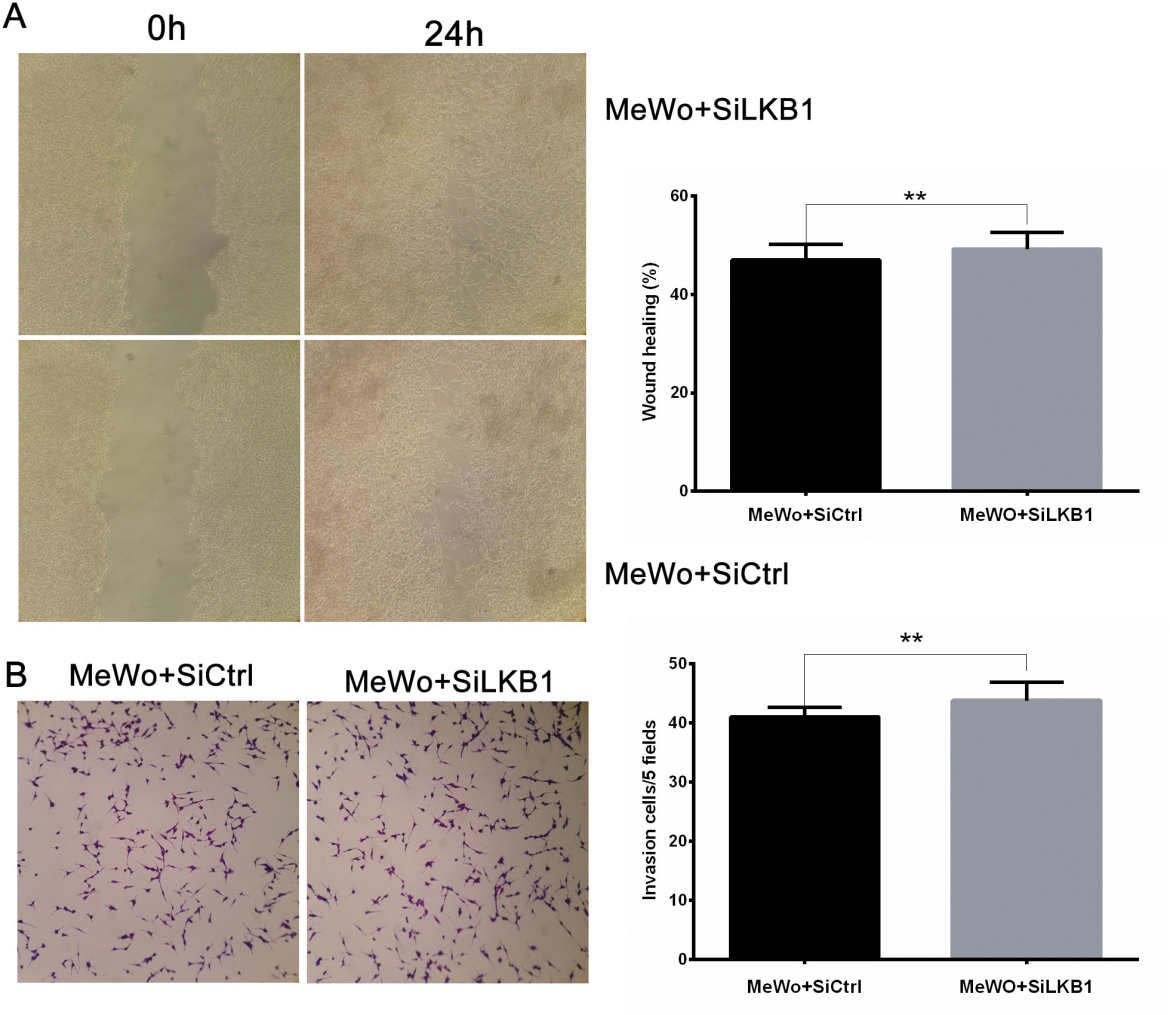
LKB1 loss alone is insufficient to influence the migration and invasion behavior of MeWo cells MeWo cells were transiently transfected with SiLKB1 and SiCtrl respectively and performed further test 3 days after transfection. **(A)** Cell migration was detected by wound scratch assay. Quantification was carried out by calculating the percentage of cell coverage to the scratch zone at 0 and 24h. Values are means±SD of triplicate experiments. **(B)** Cell invasion was tested by Transwell assay. Invaded cells were stained with crystal violet and photographed at×40 magnification. Quantification was carried by counting the number of invaded cells per 5 fields under microscope. Data are obtained from 3 independent experiments and shown as means±SD of triplicate experiments(^*^p<0.05).

Then we examined the effects of LKB1 loss on migration and invasion by performing monolayer wound healing assay and Transwell assay. As shown in Figure 2, siLKB1 group cells showed no difference with the siCtrl control group cells in migration and invasion, indicating that LKB1 knockdown did not influence the migration and invasion behavior of MeWo cells.

### LKB1 loss cooperating with BRAF V600E mutation promotes the migration and invasion behavior of BRAF V600E A375 melanoma cell lines

LKB1 loss alone does not promote melanoma cells invasion and migration, we wonder whether LKB1 silence could cooperate with other genetic events to promote melanoma progression. BRAF V600E is the most common mutation found in melanoma; then we analyzed the effect of LKB1 loss on migration and invasion of the melanoma cell lines in the presence of BRAF V600E.

**Figure 3:**
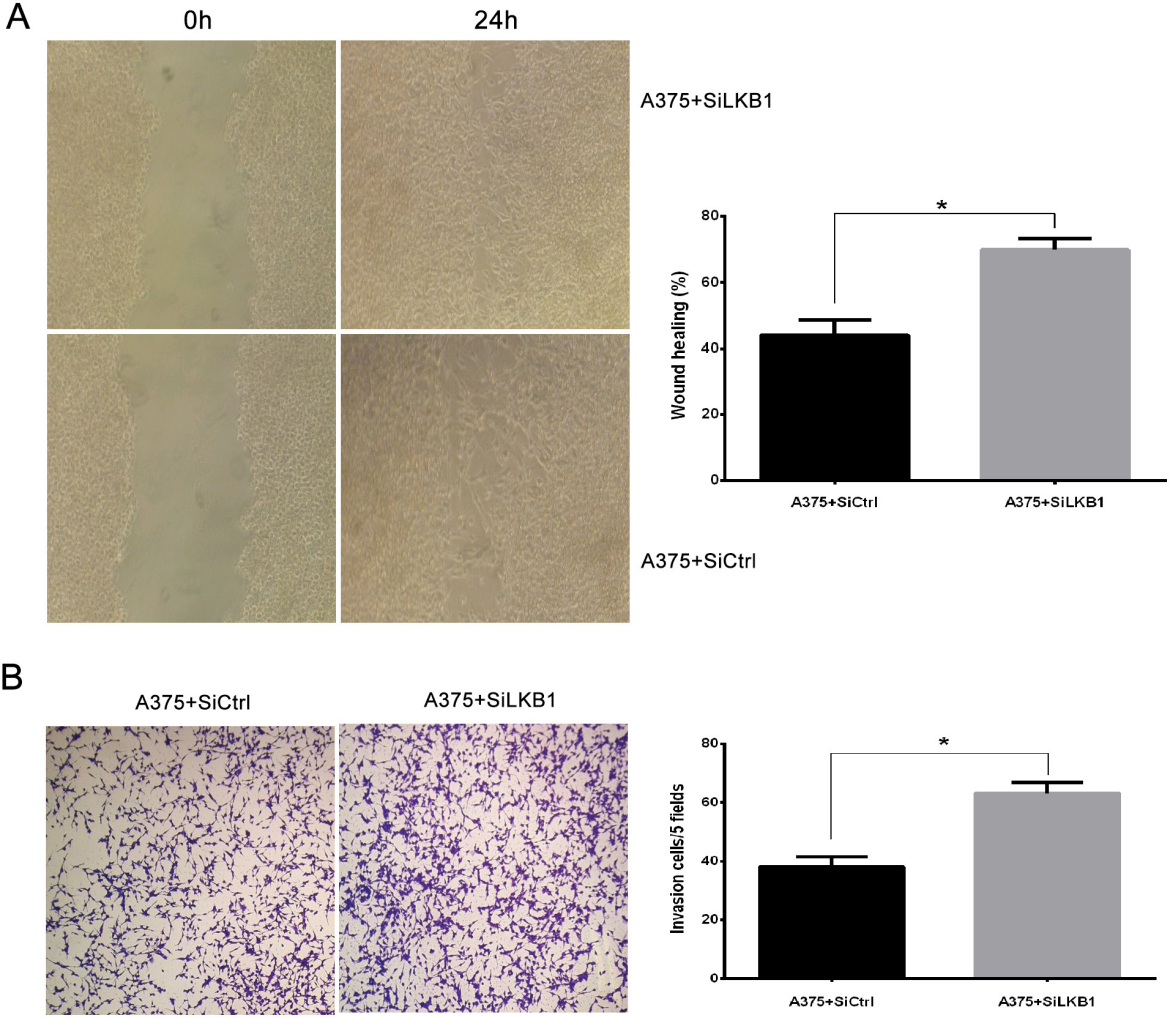
LKB1 loss cooperating with BRAF V600E mutation promotes the migration and invasion behavior of melanoma cell lines A375 cells were transiently transfected with SiLKB1 and SiCtrl respectively and performed further test 3 days after transfection. **(A)** Cell migration assay was performed according to the methods described above. A375 cells transfected with SiLKB1 showed a faster wound closure rate than the cells transfected with SiCtrl. **(B)** Cell invasion assay was performed according to the methods described above. LKB1 knockdown significantly enhanced invasion of A375 cells. Data are obtained from 3 independent experiments and shown as means±SD of triplicate experiments(^**^ p>0.05).

Similarly, LKB1 protein expression was knocked down by small interfering RNA (siLKB1) in A375 cells (BRAF V600E), A375 cells transfection with siCtrl were set as a negative control (Figure 1). Next, we examined cell migration and invasion after LKB1 inactivation. As shown in Figure 3, compared with negative control cells, LKB1 silencing remarkably enhanced A375 cells migration and invasion. According to the result of two parts, we suggested that LKB1 silencing cooperating with BRAF V600E mutation could effectively enhance the invasion and migration potentials of the melanoma cells.

### LKB1 loss in BRAF V600E melanoma cells increased cell invasion by up-regulating MMP-2 expression through the PI3K/Akt/mTOR signaling pathway

**Figure 4:**
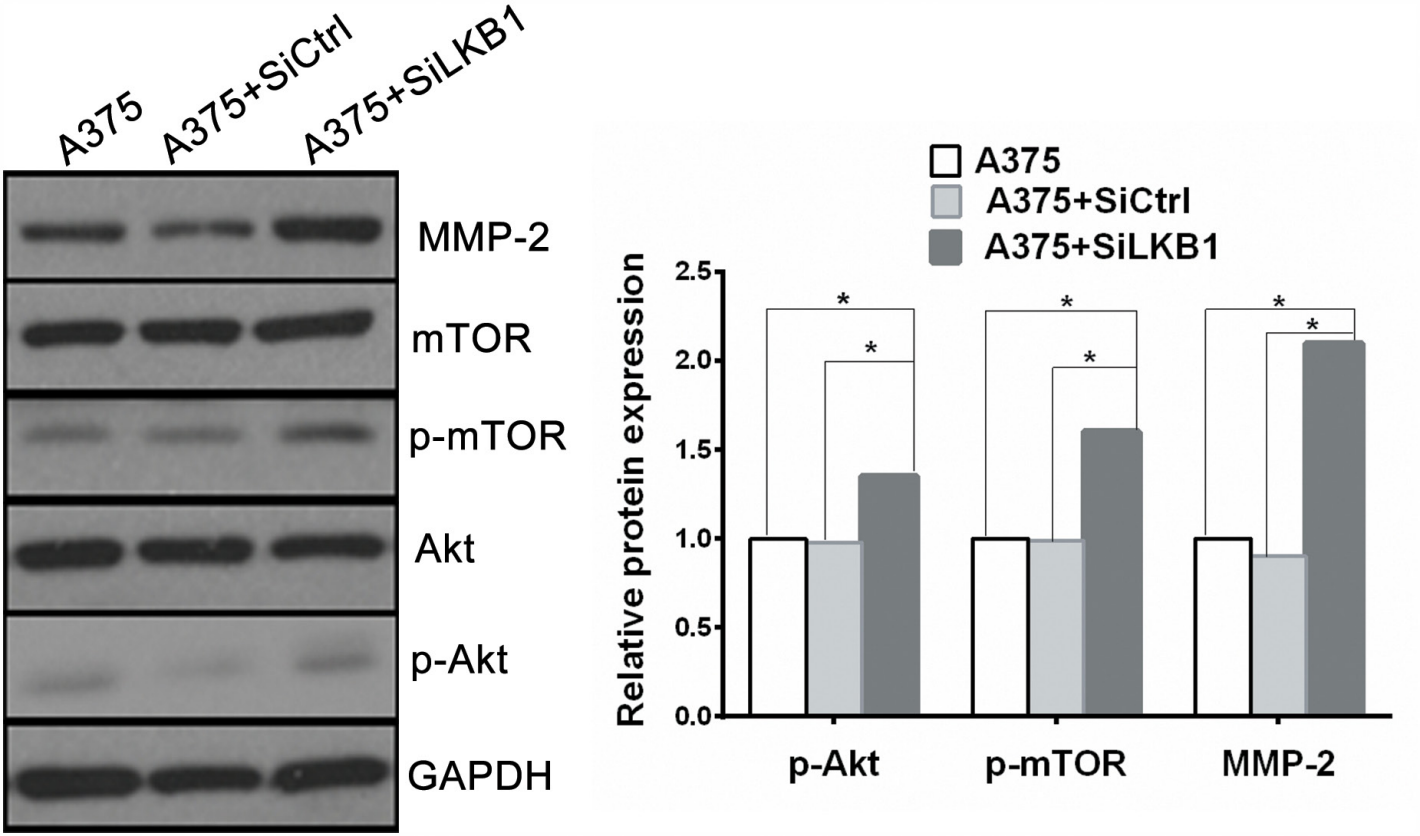
Knockdown of LKB1 expression in BRAF V600E melanoma cells increased cell invasion via up-regulating MMP2 secretion through PI3K/Akt/mTOR pathway The expression of MMP-2、phosphorylated Akt and phosphorylated mTOR were all enhanced in LKB1 knockdown A375 cells compared with A375 and A375+siCtrl cells (^*^p<0.05).

One of the key characteristics note for the melanoma cells is its high aggressive to acquire migratory and invasive capacity. Numerous studies have linked the increased invasiveness of tumors with the degradation of basement membranes and remodeling of the extracellular matrix (ECM) by proteolytic enzymes such as matrix metalloproteinase (MMPs). It has been reported that loss of LKB1 is associated with the PI3K/Akt/mTOR signaling pathway, and PI3K/Akt/mTOR signals induce the MMPs secretion. Therefore, in order to explore the potential mechanism by which LKB1 loss cooperates with BRAF V600E to promote melanoma cells invasion and migration, MMP-2 and PI3K/Akt/mTOR mediated signal molecules were investigated by using Western blot analysis in A375 cells, which transfected with siLKB1 and siCtrl respectively. As shown in Figure 4, the expression levels of MMP-2、phosphorylated Akt and phosphorylated mTOR were obviously increased in LKB1 knockdown A375 cells, but fail to significantly change in SiCtrl transfected A375 cells. We also western blotted the MMP-2 and PI3K/Akt/mTOR expressions in MeWo cells. As a result, the expression levels of MMP- 2 、phosphorylated Akt and phosphorylated mTOR were not increased in LKB1 knockdown MeWo cells as well as in SiCtrl transfected MeWo cells (Figure 6).

**Figure 5:**
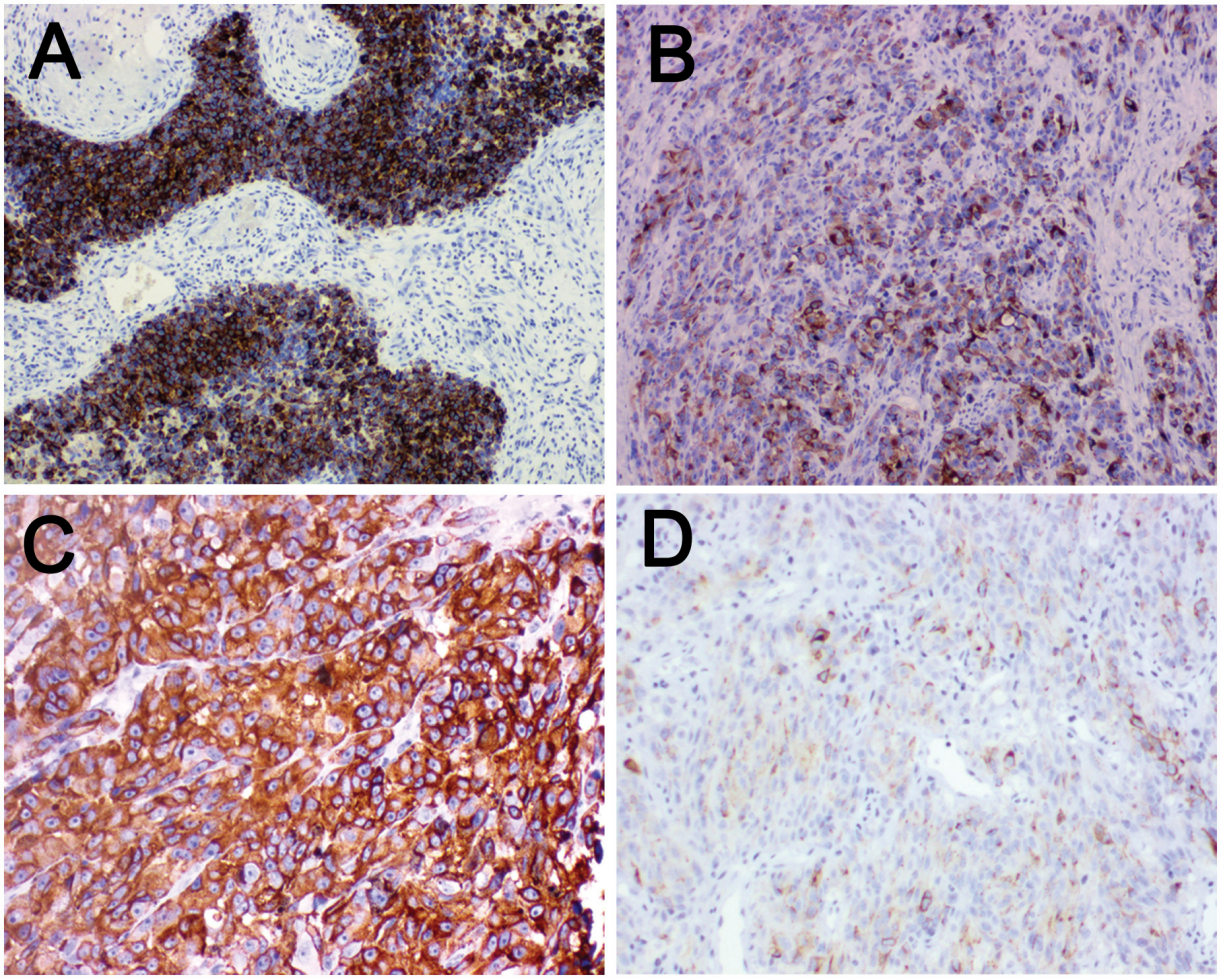
Immunohistochemical staining for LKB1 and MMP-2 in human melanoma tissue **(A)** Melanoma tissue showing LKB1high level expression at magnification×100. LKB1 expression was localized in cytoplasm. **(B)** Melanoma tissue showing LKB1 low level expression at magnification×100. **(C)** Melanoma tissue showing MMP-2 high level expression at magnification×200, MMP-2 expression was localized in cytoplasm. **(D)** Melanoma tissue showing MMP-2 low level expression at magnification×200.

**Figure 6:**
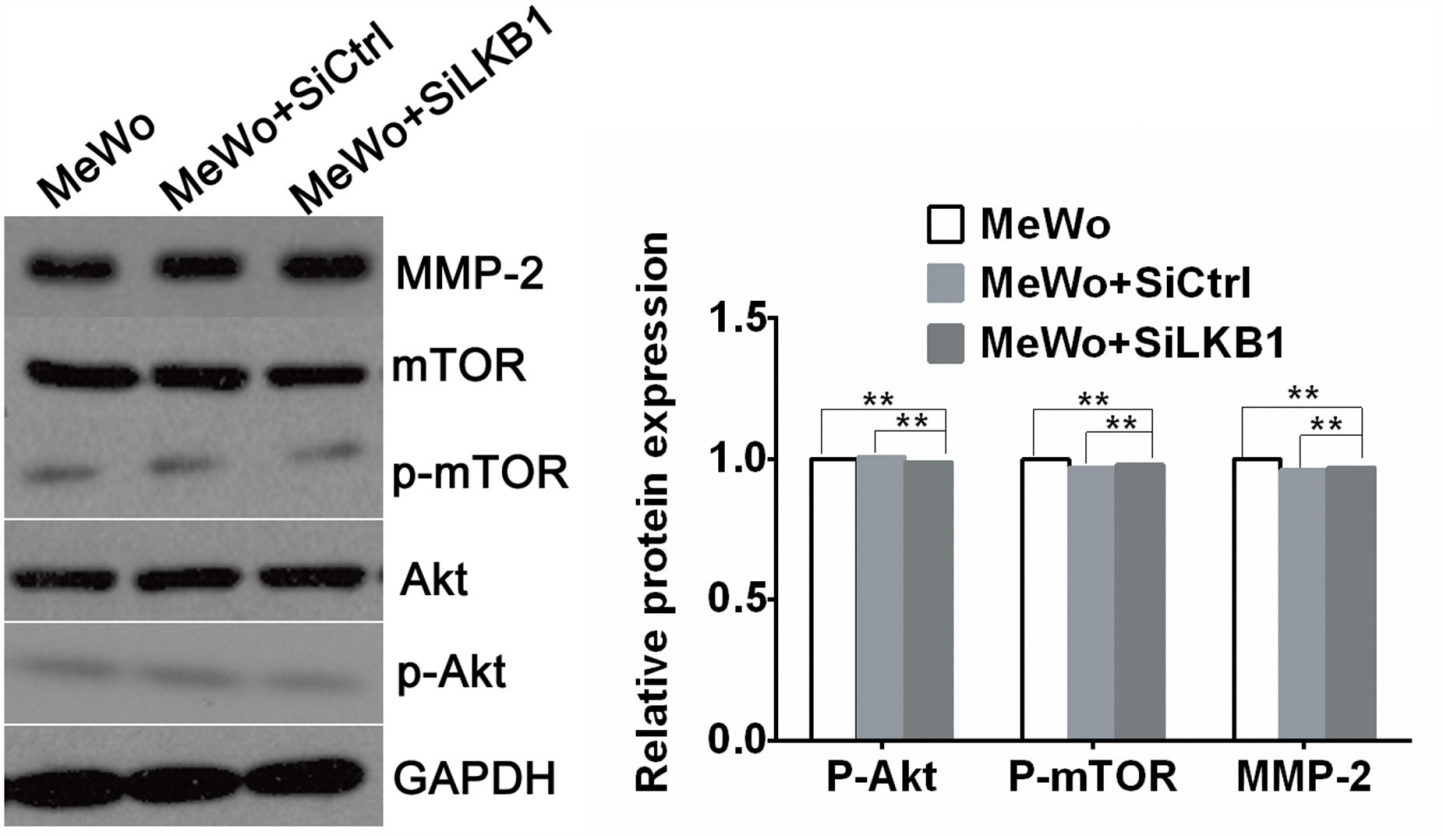
The expression of MMP-2、phosphorylated Akt and phosphorylated mTOR in LKB1 knockdown MeWo cells by western blot analysis The expression of MMP-2、p-Akt and p-mTOR in LKB1 knockdown MeWo cells showed no difference with MeWo and MeWo +siCtrl cells (^**^p>0.05). Knockdown of LKB1 in BRAF wild type MeWo cells did not increase cell invasion, nor did up-regulate MMP2 secretion through PI3K/Akt/mTOR pathway.

Taken together, our results demonstrated that PI3K/Akt/mTOR signaling pathway is involved in the migratory and invasive ability of A375 cells through up-regulating MMP-2 expression *in vitro*. This may be the underlying molecular mechanisms that LKB1 loss mediated in BRAF mutation melanoma cells.

### LKB1 low expression negatively correlated with MMP-2 expression in human BRAF V600E melanoma tissues

To further evaluate the clinical significance of LKB1 loss combined with BRAF mutation in human melanoma metastasis. We retrospectively analyzed melanoma tumors for LKB1 and MMP-2 using immunohistochemistry from 90 melanoma patients, including 27 BRAF V600E patients and 63 BRAF wild type patients, and correlated LKB1 expression with tumor clinicopathological parameters. As showed in Figure 5, LKB1 and MMP-2 expression were all localized in the cytoplasm. We observed that 49 (49/90) cases displayed LKB1 low level expression, and 41 (41/90) cases displayed LKB1 high level expression. In contrast, 35 cases exhibited a high level MMP-2 expression and 55 cases were low level or negative expression (Table 2). In general, LKB1 expression did not correlate with any clinicopathological parameter in 63 BRAF wild type cases. However, in those 27 BARF V600E cases, LKB1 low level expression was not only associated with high level MMP-2 expression (Spearman’s rank correlation test, r= -0.542, p<0.05) but also with distant metastasis and lymph node metastatic status (contingency table Chi-squared test, p<0.05) (Table 1). Overall, these results further indicate that LKB1 low levels are negatively linked to MMP-2 high levels in BRAF V600E human melanoma tissues.

**Table 1:** Correlations of LKB1 expression with different clinicopathological parameters in 90 cases of melanoma

Parameters	BRAF wild type				BRAF V600E			
	No.	LKB1 expression		p	No.	LKB1 expression		P
		Low	High			Low	High	
Age at diagnosis								
≤50	31	13	18	0.133	9	6	3	0.692
>50	32	20	12		18	10	8	
Gender								
Male	29	16	13	0.801	13	7	6	0.704
Female	34	17	17		14	9	5	
Tumor site								
Head/neck	9	5	4	0.977	4	2	2	0.655
Extremities	20	10	10		11	6	5	
Trunk	29	15	14		10	6	4	
Other	5	3	2		2	2	0	
Tumor thickness								
≤1.0mm	34	18	16	0.196	12	7	5	0.974
1.0-4.0mm	25	14	11		8	5	3	
>4.0mm	4	1	3		7	4	3	
Mitotic (×10 HPF)								
1-5	17	10	7	0.788	9	5	4	0.784
6-10	25	12	13		4	3	1	
>11	21	11	10		14	8	6	
Lymph node								
Absent	30	17	13	0.616	13	4	9	0.006^*^
Present	33	16	17		14	12	2	
Tumor subtype								
ALM	16	8	8	0.971	5	3	2	0.335
SSM	14	7	7		4	4	0	
LMM	21	11	10		6	3	3	
Mucosal	12	7	5		12	6	6	
Distant metastasis								
Absent	46	27	19	0.155	12	3	9	0.002^*^
Present	17	6	11		15	13	2	

^*^ p<0.05.

**Table 2:** The expression of LKB1 and MMP-2 in BRAF wild and mutated melanoma

	BRAF wild		BRAF mutated	
	MMP-2 high level	MMP-2 low level	MMP-2 high level	MMP-2 low level
LKB1High level	7	23	5	6
LKB1 Low level	8	25	15	1

## DISCUSSION

In this study, we observed that BRAF mutated A375 melanoma cells with LKB1 knockdown *in vitro* displayed a stronger migration and invasion ability than LKB1 intact A375 cells, while BRAF wild type MeWo melanoma cells with LKB1 knockdown did not enhanced invasion and migration ability. We postulate that the LKB1 knockdown synergism with BRAF mutation promote melanoma cells invasion and migration *in vitro*.

LKB1 is a serine/threonine kinase that functions as a tumor suppressor, which frequently inactivated in several human tumors, including lung, breast, pancreatic cancer and melanoma. Its losses are linked with tumors’ invasion and metastasis phenotypes, unfavorable clinical outcomes. However, LKB1 deficiency alone do not directly result in tumorigenesis in many human cancers, it may synergize with other gene alterations [19, 20]. For example, LKB1 haploinsufficiency were found cooperating with activated Kras in pancreatic tumorigenesis by reducing growth arrest. And in a mouse model of lung cancer, LKB1 loss was thought as a risk factor concomitant with BRAF V600E mutation leading to the tumor development. In addition, despite the successes in treatment of melanoma with BRAF inhibitors, intrinsic or acquired resistance to BRAF inhibitors still were the main problems which need to be solved. That is to say, BRAF V600E mutation status in melanoma should not be looked as the sole marker for BRAF targeted therapy [21]. Bin Zhang [16] et al recently pointed that oncogenic BRAF V600E mutant can inhibit the activity of AMPK by promoting phosphorylation of LKB1 and that this inhibition is critical for melanoma cell proliferation and growth. Based on these findings, they suggested that treatment of BRAF-mutant melanomas with a combination of a BRAF inhibitor and an AMPK activator (phenformin) could offer therapeutic advantages over BRAF inhibitor (BRAFi) single agent therapy. While Richard Marais [22] put forward a different viewpoint on treatment of BRAF mutated melanoma with AMPK activator metformin. They found that metformin whose mechanism was to inhibit the energy-sensitive LKB1-AMPK/mTOR signaling pathway and then to reduce protein synthesis and cell proliferation did not block BRAF-mutant melanoma cells growth due to the elevated protein kinase RSK activity and the increased VEGF-A protein production. Actually, the reasonable explanation for these differences was there existing molecule interaction between different signal pathways during the BRAF mutated melanoma progression.

Tumor cell invasion and migration are largely dependent on matrix degradation through matrix metalloproteinase [23-25]. Ou W [26] et al reported that LKB1 expression associated with the secretion of matrix metalloproteinases (MMPs), and MMPs could lead to the degradation of matrix surrounding the tumor cell. In addition, Chan [14] et al also found LKB1 loss in melanoma disrupts directional migration toward extracellular matrix. Similar to their researches, in our study, we investigated MMP-2、MMP-9 expression in A375 and MeWo cells respectively, our results revealed that LKB1 knockdown cooperating with BRAF mutation in melanoma may cause the activation of PI3K/Akt/mTOR signaling pathway, then promote to the secretion of MMP-2, while there had little effect on MMP-9 expression (Data not list). This may be a new explanation for the crosslink between LKB1 loss and MMPs expression.

We also retrospectively tested the LKB1 and MMP-2 expression using immunohistochemistry in formalin fixed paraffin embedding (FFPE) tissues from patients with melanoma. Similarly, BRAF V600E melanoma patients concurrent with LKB1 low expression are more likely to have lymph node metastases and related to the MMP-2 high expression. These findings are consistent with our *in vitro* studies, which demonstrated that LKB1 loss cooperating with BRAF mutation promotes melanoma cell invasion by the expression of MMPs.

In summary, our results demonstrated that LKB1 inactivation plays a cooperative role with BRAF mutation in promoting melanoma cells invasion and migration by activation of PI3K/Akt/mTOR signaling pathway and MMP-2 expression. LKB1 expression level inversely correlated with MMP-2 expression level in melanoma tissues in the presence of BRAF V600E. These finding may lead to a new therapeutic strategy against melanoma by targeting LKB1 and BRAF mutation simultaneously.

## MATERIALS AND METHODS

### Cell culture and siRNA transfection

A375 and MeWo human melanoma cell lines were purchased from KeyGEN BioTECH company and cultured in low glucose DMEM with 10% fetal bovine serum (FBS) (Gibco, US origin) at 37°Cin a 5% CO_2_ atmosphere. The targeted LKB1#1 (sense:5'-AGGGAUGCUUGAGUACGAATT-3', LKB1#2 sense:5'- ACAGAAACGAUUGUUCUACAC-3', LKB1#3sense: 5'-AAAAGGAAGGGAAAAACCCUU-3') and scrambled siRNA sequences (sense: 5'-UUCUCCGAACG UGUCACGUTT-3') were chemically synthesized. For LKB1 silencing, cells were placed in 6-well plates, seeded at a quantity of 1 × 10^5^cells, 100 nM siLKB1 and siRNA (siCtrl) were transfected into cells using Lipofectamin 2000 (Invitrogen Inc.) following manufacturer's protocol. mRNA expression detection、Western blot analysis、migration and invasion assays were performed 48 hours after transfection.

### RNA extraction and qRT-PCR

Total RNA was prepared by Trizol extraction (KeyGEN BioTECH, KGA1202, China) and reverse transcribed into cDNA using M-MLV reverse transcriptase (KeyGEN BioTECH, KGA1307, China) according to the manufacturer’s protocol. Real-time reverse transcription-PCR was performed with Maxima SYBR Green Real-time PCR (KeyGEN BioTECH, KGA1339, China). Human LKB1 primers were synthesized based on the published sequences 5-AGGGATGCTTGAGTACGAACC-3(forward) and 5-GTCCTCCAAGTACGGCACC-3 (reverse). LKB1 mRNA relative expression was analyzed using the 2ΔCt method with GAPDH as an internal normalization control.

### Western blot analysis

Total cell lysates and precipitates were collected and separated by SDS-PAGE, then transferred onto a nitrocellulose membrane. Western blotting was performed as previously described. The membranes were incubated with primary antibodies against LKB1 (ab185734, Abcam), MMP-2 (ab37150, Abcam), Akt (ab8805, Abcam) and phospho-Akt (phospho T308, ab38449, Abcam), mTOR (ab32028, Abcam), phospho-mTOR ((phospho S2448, ab84400, Abcam) overnight at 4°C followed by HRP-conjugated secondary antibodies (Invitrogen, Catalog#: 31460) for 1hour at room temperature. Afterwards, band images were detected using ECL reagent (Amersham Biosciences). The experiment was repeated 3 times.

### Wound scratch assay

Wound scratch assay was performed as described previously. Transfection and control cells were grown to confluence in a 6-well plate respectively. After 24 hours, cells were wounded using sterile pipette tip. The wounded area was photographed at each time courses at 0 and 24hours. The wound healing capabilities were calculated by counting the percentage of cell coverage to the scratch zone at 0 and 24h. Mean values were obtained from at least 3 separate experiments.

### Transwell migration assay

Invasion of transfection and control cells (2.5×10^4^ cells per well) were measured using 24-well Matrigel invasion chamber kits (BD Biosciences, Bedford, MA, USA) according to the manufacturer’s instructions. Cultured cells were plated at 1 × 10^5^ per well into the upper transwell chambers and 20% FBS-containing medium was placed into the bottom chamber. After incubation at 37°C in 5% CO_2_ for 12h, the cells that invaded through the 8-mm sized pores and adhered to the lower surface of the membrane were fixed with 4% paraformaldehyde, stained with crystal violet and counted in at least five random fields of view (×40) for each well.

### Tissue specimens mutation screening and immunohistochemistry

Formalin-fixed paraffin embedded (FFPE) melanoma archival tissue blocks were from the first affiliated hospital of Nanjing medical university, China, between 2012 and 2015. All tissues were firstly subjected to BRAF V600E mutation analysis by using AmoyDx™ BRAF V600E Mutation Detection kit (Amoy Diagnostics, Xiamen, China) according to the manufacturer's instructions, then to immunohistochemistry staining according to the routine operation procedure reported in previously study. Patients’ name and other involved privacy were hidden during the whole study process. The ethics committee of the hospital approved our research design, including the use of all tissue. Rabbit anti–human LKB1 Primary antibody (1:250) and MMP-2 (1:200) were purchased from abcam Company. Two pathologists who are blind to this research reviewed all tissue sections respectively and made the consistent interpretation of IHC results based on the percent of positively stained cells and the staining intensity. Briefly, the percentage of positive staining was scored as 0 (0%), 1 (0%–20%), 2 (20%–50%) or 3 (over 50%), and the intensity as 0 (no staining), 1 (weak staining, visible at high magnification), 2 (moderate staining, visible at low magnification) and 3 (dark staining, strikingly positive at low magnification). The total immunostaining score was calculated with the value of percent positivity score multiply staining intensity score, which ranged from 0 to 9. The expression level of LKB1 、MMP-2 was defined as low expression group (score 0-3) and high expression group (score 4-9).

### Statistical analysis

Statistical analyses were performed using SPSS19.0 (SPSS Inc., Chicago, Illinois, USA) software. Comparisons of LKB1 expression with clinicopathological characteristics were made using contingency table Chi-squared test. The significant correlations between the expression of LKB1 and MMP-2 were calculated using spearman’s correlation analysis. All p values are two-sided and considered statistically significant at the 0.05 level.
